# Effectiveness of interventions to promote healthy diet in primary care: systematic review and meta-analysis of randomised controlled trials

**DOI:** 10.1186/1471-2458-13-1203

**Published:** 2013-12-20

**Authors:** Nawaraj Bhattarai, A Toby Prevost, Alison J Wright, Judith Charlton, Caroline Rudisill, Martin C Gulliford

**Affiliations:** 1 Department of Primary Care and Public Health Sciences, King’s College London, London, UK; 2 Department of Social Policy, London School of Economics and Political Science, London, UK; 3 Health Economics and Health Technology Assessment Unit, Institute of Health and Wellbeing, University of Glasgow, 1 Lilybank Gardens, Glasgow G12 8RZ, UK

**Keywords:** Diet, Health promotion, Primary care, Systematic review, Meta-analysis

## Abstract

**Background:**

A diet rich in fruit, vegetables and dietary fibre and low in fat is associated with reduced risk of chronic disease. This review aimed to estimate the effectiveness of interventions to promote healthy diet for primary prevention among participants attending primary care.

**Methods:**

A systematic review of trials using individual or cluster randomisation of interventions delivered in primary care to promote dietary change over 12 months in healthy participants free from chronic disease or defined high risk states. Outcomes were change in fruit and vegetable intake, consumption of total fat and fibre and changes in serum cholesterol concentration.

**Results:**

Ten studies were included with 12,414 participants. The design and delivery of interventions were diverse with respect to grounding in behavioural theory and intervention intensity. A meta-analysis of three studies showed an increase in fruit consumption of 0.25 (0.01 to 0.49) servings per day, with an increase in vegetable consumption of 0.25 (0.06 to 0.44) serving per day. A further three studies that reported on fruit and vegetable consumption together showed a pooled increment of 0.50 (0.13 to 0.87) servings per day. The pooled effect on consumption of dietary fibre, from four studies, was estimated to be 1.97 (0.43 to 3.52) gm fibre per day. Data from five studies showed a mean decrease in total fat intake of 5.2% of total energy (1.5 to 8.8%). Data from three studies showed a mean decrease in serum cholesterol of 0.10 (-0.19 to 0.00) mmol/L.

**Conclusion:**

Presently-reported interventions to promote healthy diet for primary prevention in primary care, which illustrate a diverse range of intervention methods, may yield small beneficial changes in consumption of fruit, vegetables, fibre and fat over 12 months. The present results do not exclude the possibility that more effective intervention strategies might be developed.

## Background

An increase in intake of fruit and vegetables of one portion per day (80 g/day) may be associated with a 10% relative reduction in risk of ischaemic heart disease and 6% reduction in stroke, with between 1% and 6% reduction in risk of certain cancers [1]. However, a typical American diet includes only includes 42% of the recommended daily intake of fruit, and 59% of the recommended intake of vegetables [2]. A higher intake of dietary fibre is associated with lower risk of all-cause mortality [3], as well as lower incidence of colorectal cancer [4] and stroke [5]. The estimated mean fibre intake for American adults is 15.9 gram per day, lower than the recommended intake of at least 25–38 gram per day [6]. Cardiovascular diseases and diabetes are associated with obesity and high dietary intakes of fat and sugars [7] but a typical American diet includes 280% of the recommended intake of calories from solid fats and sugars [2]. Obesity imposes a significant burden of morbidity and mortality on populations. The health care costs associated with obesity are substantial and the vast majority of the costs are attributable to treating health consequences of obesity including type 2 diabetes, cancer and cardiovascular diseases [8].

There is evidence for the effectiveness of primary care-based interventions to promote physical activity [9], alcohol reduction [10] and smoking cessation [11]. The regularity of patient consultations in primary care [12], and the value that patients place on medical advice [13], offer opportunities for general practitioners to play important roles in promoting health and preventing disease. This may include the provision of advice on healthy eating. Several randomised trials have evaluated the potential to modify patients dietary habits through primary care based interventions. However, earlier systematic reviews of the effectiveness of dietary interventions for primary prevention are limited in their applicability to primary care by the inclusion of studies set in work places, shopping centres and churches. Some reviews have included non-randomised studies [14] and trials with short follow-up, as well as participants with established medical conditions, or patients with defined high-risk status [14-17], with the possibility of diet restrictions and which would limit the participation in diet promotion intervention.

Recent work on understanding the effectiveness of interventions to increase healthy diet has focused on the importance of using behaviour science theory to understand determinants of behaviour. Use of specific intervention techniques including setting goals, monitoring behaviour, and reviewing progress towards goals in the light of feedback may be key to dietary behaviour change [18,19]. The effectiveness of behavioural interventions may also depend on factors such as the frequency of contacts, the type of professional involved, and whether delivered individually or in a group setting.

We report a systematic review and meta-analysis of randomised controlled trials of primary care-based diet promotion interventions for primary prevention in adults with minimum 12 months follow up. The aim was to quantify whether diet promotion for primary prevention in primary care is effective in sustained dietary modifications over at least one year. We also aimed to characterise existing interventions in terms of their theoretical basis and intervention techniques employed, and explore whether these were related to intervention effectiveness.

## Methods

### Eligibility criteria

The review included reports of randomised or cluster controlled trial study designs. *Outcome measures* included fruit and vegetable intake (servings/day), fat (% of total energy intake), fibre consumption (gram per day) and change in serum cholesterol level (mg/dl or mmol/l). *Interventions* included any diet promotion intervention in primary care, including dietary counselling, motivational interviews, advice for behaviour change, computer-delivered dietary information, reminder telephone calls and postal newsletters. Primary care in this context refers to interventions delivered through the first point of contact in a health care system, where the service provider acts as the principal source of advice to patients, rather than through specialist referral. Dietary promotion intervention in this context means any methods which are used to promote healthy diet, including healthy eating advice and counselling, telephone calls, group lectures or use of any other dietary education materials including posters, booklets and guidelines. We excluded multifaceted interventions including those with physical activity promotion along with diet promotion and we did not set any threshold amount of physical activity for exclusion. *Target populations* included the general population of adults aged 16 years or over, including both men and women. We excluded studies in pregnant women, patients at high risk of, or diagnosed with, cardiovascular diseases, type 2 diabetes, cancer or other chronic conditions, as well as studies in first or second degree relatives of affected individuals. We also excluded studies that included participants at high risk of colorectal cancer (because of adenomatous polyps) [20] or breast cancer (with mammographic abnormalities) [21,22]. In order to focus on a population approach to primary prevention, we excluded trials which included participants who were pregnant, or with existing chronic conditions, or at high risk of diseases such as colorectal or breast cancer, or with participants who were relatives of family members with chronic health problems linked to diet. Such high risk participants or those with established chronic conditions linked with diet, may be more motivated to make dietary behaviour changes which may apparently show higher effectiveness of interventions promoting healthy diet in primary care. Also, there may be possibilities of diet restrictions which may limit the participation in diet promotion intervention and may apparently show lower effectiveness. *Comparators* included usual care or no intervention. We excluded those trials comparing one type of diet promotion intervention with another only because our aim was to estimate the effect size difference between a diet promotion intervention and the existing usual care or no intervention; we did not aim to compare any two methods of diet promotion interventions. A minimum *follow- up period* of 12 months after randomisation was required. Only English language publications were included.

### Search methods, study selection and data extraction

We searched Medline, PsycINFO, EMBASE, Centre for Reviews and Dissemination, and the Cochrane Library, with no restrictions in the date and year, using the combined search terms “dietary intervention AND primary care”, “diet promotion intervention AND primary care”, “diet advice AND primary Care”, “counselling AND diet AND primary care”, “diet promotion AND primary care”, “diet advice AND behaviour change”, “advice in primary care AND behaviour AND diet”, “nutritional counselling AND primary care”, “lifestyle counselling AND cardiovascular risk AND primary care”, “fruit AND vegetable AND primary care”, “nutritional counselling AND general practice”, “dietary intervention AND general practice”, “dietary intervention AND primary care practice”, “nutritional advice AND general practice”, “primary care and diet modification”, “fruit and vegetables consumption AND general practice” “primary care AND fiber consumption” and “fruit and vegetable consumption AND diet intervention”. We also reviewed reference lists of relevant articles and previous systematic reviews. The search was carried out initially in September 2012 and again in March 2013. NB carried out initial screening of title and abstracts against inclusion criteria and retrieved those potentially eligible. NB and MCG independently assessed the retrieved full text articles and any differences were reviewed and agreed. NB extracted data concerning participants, interventions, and outcomes in a tabular form designed for this review. AJW and NB extracted data on the nature of each intervention, including total number of contacts with participants, mode(s) of administration and intervention techniques used [23] and coded the extent to which intervention was based on psychological theories of the determinants of behaviour change, using a published coding scheme [24]. NB and MCG cross checked the extracted data.

### Methodological quality assessment

NB appraised each study for methodological quality using a standard guidance and checklist [25]. We assessed the methodological quality and risk of bias in terms of randomisation, allocation concealment, blinding, loss to follow up and outcome assessment tool validity.

### Statistical analysis

For each trial, we extracted the intervention effects at 12 months. We estimated the intervention effect as the difference in the change in mean outcome values (follow up value minus baseline value) between the intervention group and control group. If the baseline data were not reported in the trials, we used the difference in the mean outcomes between groups at follow up. If not supplied, we followed standard procedure [26] to derive standard error (SE) for each measure. Where fruit and vegetable consumption was expressed in grams, the conversion factor of one serving = 80 gm was used to express them as servings and where the serum cholesterol was expressed in mg/dl, the conversion factor of 38.6598 was used to express them as mmol/L.

We used random effects meta-analysis to pool the estimates from individual studies. We used the I^2^ statistic to describe the variation in effect size attributable to heterogeneity among studies; higher values suggesting greater heterogeneity. We also constructed funnel plots to assess for the publication bias for the studies included in the review. We used the metan command in STATA version 12 for the analysis.

## Results

### Identification of trials

**Figure 1:**
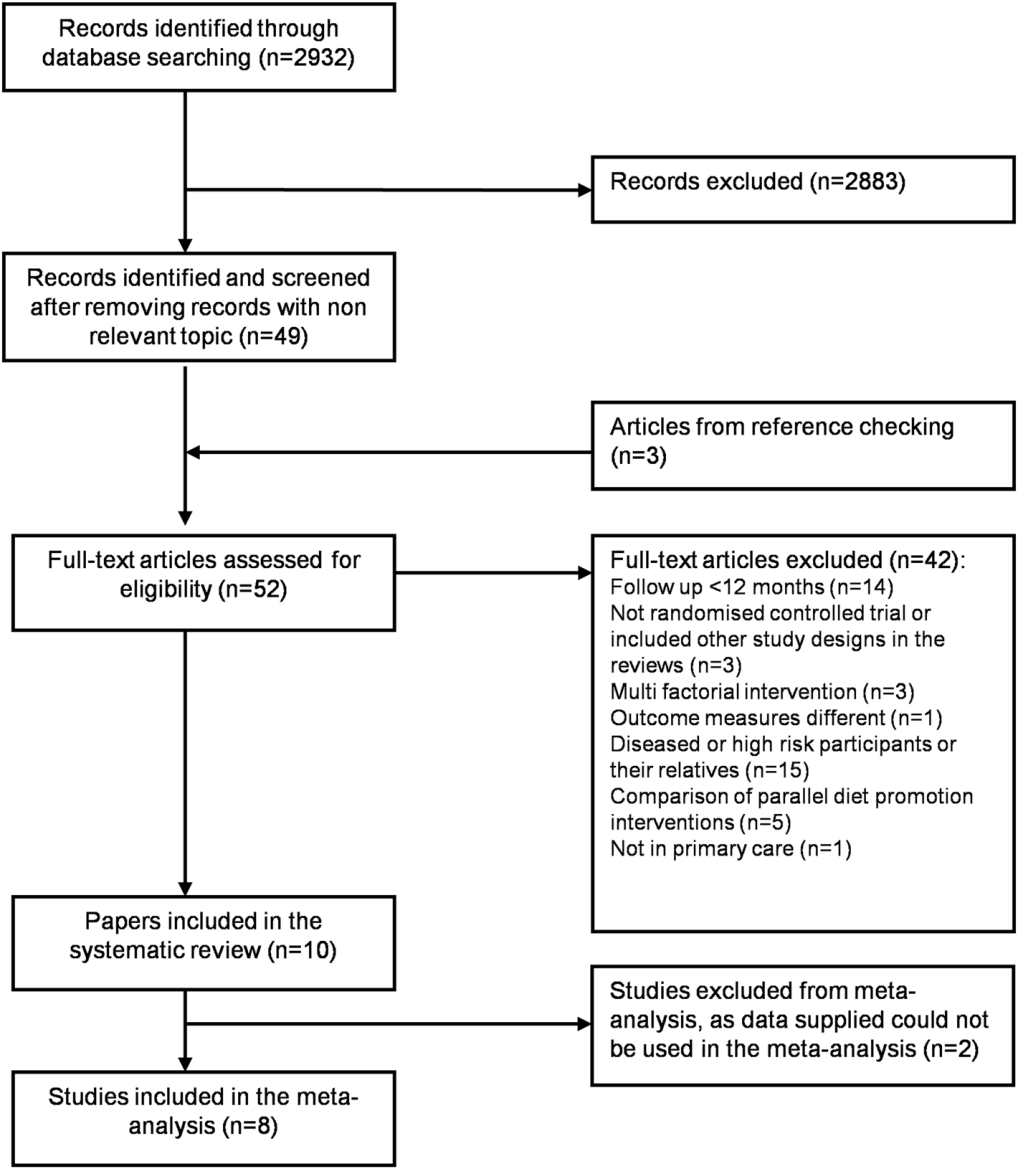
Study selection flow diagram.

We screened titles and abstracts of 2,932 papers and identified 49 full text articles. We further identified 3 full text articles from cross-checking references of 49 full text articles. We then identified 10 trials [27-36] for inclusion in this study after excluding trials not meeting the eligibility criteria. The details are shown in the flow diagram (Figure 1).

### Study and participants characteristics

We present the study and participant characteristics in Table 1. Ten studies included in the systematic review were published between 1988 and 2006. These studies were conducted in samples representing the primary care general population in Japan [27] (1 study), USA [28,31,32,34-36] (6 studies), Italy [30] (1 study) and UK [29,33] (2 studies).The randomised study sample size varied among studies and ranged from 213 to 3,179 participants with 12,414 participants randomised in total.

**Table 1:** Study and participant characteristics

**Study (Year)**	**Country**	**Study design**	**Selection of participants**	**Number of practices**	**Participants randomised (% Female)**	**Eligible age range (mean) years**	**Ethnicity and socioeconomic status**	**Diet assessment tool**
Baron (1990) [33]	UK	RCT	Randomly selected participants registered with a family practice	One group general practice	437 randomised 368 participated (49)	25-60 (41.7)	Social class 1 or 2: controls, 30% men, 24% women; intervention 39% men, 43% women.	Self-administered food frequency questionnaire
Beresford (1997) [35]	USA	Cluster RCT	Participants attending routine visits without major illness	28 physician practices within 6 clinics	2121 (68)	26% > 65 years	White: 91%; Some college education: 73%. Family income below $25000 per year: 28%.	Telephone interview administered food frequency questionnaire
Coates (1999) [28]	USA	RCT	Post menopausal women volunteers, consuming at least 36% energy from fat	University clinical centres in three states	2208 (100)	50-79 (60)	White (55%), Black (28%), Hispanic (16%); <High School (11%), High School (20%), Post high school with no college degree (35%), graduate /post graduate (33%)	Self-administered food frequency questionnaire
Fries (2005) [34]	USA	RCT	Randomly selected participants from physicians’ lists	Three rural Virginia physician practices	754 (64)	18-72 (46.34)	White: 61%, African American: 37%; 8th grade: College degree: 24%; Income < $10,000: 14.69%, ≥$41,000:19%.	Telephone interview administered fat and fibre behaviour questionnaire
Gann (2003) [36]	USA	RCT	Women volunteers aged 20–40 years recruited through advertising and direct mail in Chicago.	One clinic	213 (100)	20-40 (33.4)	76% White, 13.5% Black, 4% Hispanic, 5.5% Asian, 1.5% other; 85% completed college	Telephone interview administered food frequency questionnaire, based on 24 hr diet recall on each of three days
Kristal (2000) [32]	USA	RCT	Randomly selected patients enrolled with an HMO.	Health maintenance organisation	1459 (50)	18-69 (45.8)	White (85.9%), Black (4.5%), Asian (5.8%), Hispanic (3.0%), Other (0.8%); Household income < $25,000 12.2%, ≥$70,000 21.7%.	Telephone interview administered Food Frequency Questionnaire (FFQ) and Diet Habits Questionnaire
Roderick (1997) [29]	UK	Cluster RCT	Unselected patients attending GP surgery practices	8 family practices	956 (50)	35-59 (47.3)	Non-manual occupation, intervention 60%, control 49%; rented accommodation intervention 11%, control 25%.	Self-administered food frequency questionnaire.
Sacerdote (2006) [30]	Italy	RCT	Unselected patients, not obese, no chronic disease	33 general practitioners	3179 (50)	18-65 (44.5)	Not reported	Family physician administered food frequency questionnaire
Stevens (2003) [31]	USA	RCT	Women with recent negative mammogram and total cholesterol ≥200 mg/dl	Health maintenance organization (HMO)	616 (100)	40-70 (53.8)	Minority groups: 7%; College graduates: 40%	Self-administered fat and fibre behaviour questionnaire (FFBQ)
Takahashi (2006) [27]	Japan	RCT	Healthy volunteers in two rural villages, advice given after annual health checks	Not reported	550 (68)	40-69 (56)	Not reported	Self-administered diet history questionnaire (DHQ)

Participants were men and women, but three studies [28,31,36] included women only, with the age ranging from 18 to 79 years. Participants were generally healthy without established chronic diseases. Participants in one study [28] were postmenopausal women consuming at least 36% energy from fat; participants in another study [31] had serum cholesterol values of 200 mg/dl (5.17 mmol/l) or more but as this is close to the population mean value, this study was not excluded as being directed at high risk individuals.

### Intervention and control characteristics

The diet promotion interventions in the trials varied in number of contacts with participants, mode of delivery and behaviour change techniques employed (Table 2).The number of scheduled contacts for intervention with the participants in the intervention groups ranged between one and twenty. Most involved at least one face to face contact, but two [32,34] involved only a combination of telephone calls and mailed intervention materials. Of the interventions using face-to-face sessions, only one [28] was solely delivered in a group format, while the others used a combination of group and individual contacts. Many of the interventions also involve printed materials. The interventions involved between two and eight intervention techniques. Interventions that involved a greater number of contacts with participants did not necessarily employ a greater number of techniques.

**Table 2:** Intervention characteristics

**Study (Year)**	**Mode(s) of administration**	**Intervention intensity**	**Stated theoretical approach**	**Use of theory**	Intervention techniques used*	**Total techniques used**	**CT techniques used**	**Control condition**
Baron (1990) [33]	Face to face, individually or in small groups, supported by booklet, delivered by nurses.	Dietary advice and a booklet with advice on diet, promotional materials displayed at the practice. 30 min per session, individually or in groups, brief follow up sessions were scheduled at one and three months after entry into the study	none	N/A	1. provide information on consequences of the behaviour	3	0	No dietary advice
					21. Provide instruction on how to perform the behaviour			
					27. use of follow-up prompts			
Beresford (1997) [35]	1) Face to face - physician introduces self-help booklet;	Self-help booklet and physician endorsement to promote dietary change such as improving health, following the changing social norm to eat lower fat, higher fibre foods, and doing something positive for oneself. Introduction of booklet taking less than 3 minutes, 2 weeks later, a reminder letter signed by physician sent to the participants who had received the intervention.	Social learning theory	No	1. provide information on consequences of behaviour	8	2	No intervention/ usual care
					3. provide information regarding others’ approval			
	2) mailed reminder letter							
					5. goal setting (behaviour)			
					8. Barrier identification and problem solving?			
					9. Set graded tasks			
					19. Provide feedback on performance			
					21. provide information on how to do the behaviour			
					27. Use of follow up prompts			
Coates (1999) [28]	Face to face, in groups, delivered by nutritionists	Dietary counselling sessions in groups that met weekly for 6 weeks, bi-weekly for 6 weeks, monthly for 9 months and then quarterly until 18 months. Group members shared experiences.	None	N/A	5. goal setting – behaviour	8	2	Not counselled, but given *Dietary Guidelines for Americans*
					8. problem solving			
					12. prompt rewards contingent on effort/success towards behaviour and on successful behaviour			
					16. prompt self monitoring			
					21. provide information on how to perform the behaviour			
					22. model/demonstrate the behaviour			
					26. prompt practice			
					29. plan social support			
Fries(2005) [34]	Mail plus one phone call – no information on the professional group (if any) of staff making the phone call	Intervention by telephone and mail. Including personalized dietary feedback, low-literacy self-help booklets. Phone call 2 weeks after the personalised dietary feedback with brief counselling. Information booklet: mailed in staggered format, one each week immediately after the intervention phone call.	Community-based social marketing, social cognitive theory, TTM	Yes – stage of change from the TTM	5. goal setting – behaviour	8	2	No intervention
					8. problem solving			
					12 prompt rewards contingent on effort/success towards behaviour and on successful behaviour			
					16. prompt self monitoring			
					21. provide information on how to perform the behaviour			
					22. model/demonstrate the behaviour			
					26. prompt practice			
					29 plan social support			
Gann (2003) [36]	Face to face - group sessions plus two individual sessions – no information on the professional group (if any) of staff delivering the sessions	Classroom nutrition education plus individual counselling with 18 group classes and 2 individual meetings in 12 months. To maximize the impact of intervention, appropriate foods and meals were prepared and served at intervention sessions to reinforce new eating behaviours and demonstrate the ease of preparations. Sessions included discussion and practice of shopping, label reading, and meal preparation techniques, eating out and convenience foods	None	N/A	21. provide information on how to perform the behaviour	2	0	No intervention until after end of study
					22. model/demonstrate the behaviour			
Kristal (2000) [32]	Mail plus one phone call delivered by a “trained health educator”	Tailored dietary intervention including i) a package of self-help materials, ii) dietary analysis with behavioural feedback, iii) a motivational phone call, and iv) 'semi-monthly’ newsletters.	Social learning theory, TTM, diet individuation model	Yes – intervention tailored to stage of change, motives for changing diet and stated interest in dietary change	1. provide information about the consequences of the behaviour	7	2	Usual care (No intervention)
					5. goal setting – behaviour			
					9. set graded tasks			
					19. provide feedback on behaviour			
					21. instruction on how to perform the behaviour			
					22. model/demonstrate the behaviour (?)			
					27. use of follow-up prompts			
Roderick (1997) [29]	Face to face – individual sessions, delivered by nurses plus two “further assessment” sessions delivered by GP if CVD risk factors elevated	Dietary advice aimed for food substitution after the review of the type, quantity and frequency of key foods consumed. Specially designed dietary sheets were given out. Review at second visit. 3 and 6 month reviews and GP referral if cardiovascular risk factors elevated.	None	N/A	5. goal setting- behaviour	3 or 4***	2 or 3**	Standard health education leaflet, *Guide to healthy eating*
					10 prompt review of behavioural goals			
					16. or 17.(For some) self-monitoring – not quite clear if this was of the behaviour or of weight.			
					21. instruction on how to perform the behaviour			
Sacerdote (2006) [30]	Face to face – individual session, delivered by GP, supported by booklet	Personalised nutritional intervention, based on a brochure about diet and health that summarized the Italian Guidelines for a Correct Nutrition 1998 and on a 15 min educational intervention, 2 follow-up visits to the GP.	None	N/A	1. provide information about the consequences of behaviour	2	0	A simpler and non personalized conversation without the use of a brochure.
					21. provide instruction on how to perform the behaviour			
Stevens (2003) [31]	Face to face – individual sessions plus phone calls delivered by master’s degree level health counsellors, supported by print materials	Individual 45 minute counselling sessions and telephone support. Print out of the counselling session along with nutrition education materials including descriptions of the desired dietary pattern and advice. Second 45 minute visit, 2–3 weeks after the first.	Social cognitive theory, TTM	Yes – personal barriers, self efficacy and stage of change	5. goal setting – behaviour	7	3	No dietary advice, however advised on Breast Self Examination(BSE)
					8. barrier identification and problem solving			
					9. set graded tasks			
					10. prompt review of behavioural goals			
					19. provide feedback on performance			
					21. provide instruction on how to perform the behaviour			
					37. motivational interviewing			
Takahashi (2006) [27]	Face to face, individual sessions plus one group session, postal newsletters. Professional group of those delivering the intervention unclear	Two 15 min dietary counselling sessions, a group lecture and two newsletters	None	N/A	5. goal setting – behaviour	3	2	No intervention
					19. provide feedback on performance			
					21. provide instruction on how to do the behaviour			

Key: CT = control theory, TTM = transtheoretical model.

*coded using CALO-RE taxonomy [23]; **Number of intervention techniques used consistent with control theory (out of the following four intervention techniques: prompt specific goal setting, prompt review of behavioural goals, prompt self monitoring of behaviour and provide feedback on performance); ***4 techniques used if participant was overweight, otherwise three techniques.

Four [31,32,34,35] of the ten interventions were explicitly described as being based on at least one psychological theory of behaviour change. While none of the four reports explicitly linked all components of the intervention to all the relevant constructs of the theoretical model(s) upon which they claimed to be based, they all explicitly linked at least one intervention technique to at least one determinant of behaviour specified by relevant psychological theory. In three [31,32,34] of the interventions, the intervention was tailored for participants according to how they varied on a psychological construct specified by a theory.

In each study, the control group was not enrolled in any intervention, but four had minimal interventions: dietary guidelines [28], standard health education from leaflets [29], a non-personalised conversation without diet counselling [30] and breast self-examination counselling [31] (Table 2).

In each trial, previously validated self-administered food frequency questionnaires, or modified simpler versions, were used to measure the study outcomes. Diet intakes were estimated using the average of the diet consumption in the previous 24 hours to 1 month, collected using food frequency questionnaires.

### Methodological quality of included studies

The funnel plots and Egger’s test for potential publication bias were not informative as insufficient studies were identified for each outcome. Table 3 presents a summary of the methodological quality of included studies. In general, description of randomisation method, blinding of the outcome assessment and allocation concealment were poor in the trials. None of the studies reported what steps were taken to ensure intervention fidelity [37]. Only one study assessed the psychological constructs targeted by the intervention, and briefly reported a mediation analysis to see if the intervention worked by changing the targeted beliefs. Therefore, the ability of the studies to test the psychological mechanisms of the interventions’ effects on dietary intakes was seriously compromised [24].

**Table 3:** Risk of bias assessment for included studies

**Study (Year)**	**Randomisation method**	**Allocation concealment**	**Blinding**	**Participation at 12 months**	**Outcome assessment validity reported**	**Intention to treat (ITT) analysis**
Takahashi (2006) [27]	Random numbers generated in Excel	Not stated	Partial. Nurse assessment was blinded	448/550 (81%)	Yes	No
Coates (1999) [28]	Block randomisation	Not stated	Not stated	1,141/2,208 (52%)	Yes	No
Roderick (1997) [29]	Pairs matched by region	Not stated	Not stated	Intervention 86%; control 74%	Yes	Yes
Sacerdote (2006) [30]	Random numbers generated by computer	Yes	Outcome assessors and participants stated to be blinded	2,977/3,179 (93%)	Yes	Yes
Stevens (2003) [31]	Not stated	Not stated	Partial. Clinic staff conducting data collection were blinded	Intervention 89%; control 85%	Yes	No
Kristal (2000) [32]	Stratified by sex and age	Not stated	Not stated	1,205/1,459 (83%)	Partial	No
Baron (1990) [33]	Not stated	Not stated	Not stated	329/368 (89%)	Not stated	No
Fries (2005) [34]	Not stated	Not stated	Not stated	516/754 (68%)	Yes	No
Beresford (1997) [35]	Table of random numbers	Recruiters and potential participants blind to group allocation	No	1,818/2,121 (86%)	Yes	No
Gann (2003) [36]	Table of random numbers	Not stated	Not stated	177/213 (83%)	Yes	Yes

### Effects of Intervention

#### Fruit and vegetable intake

**Figure 2:**
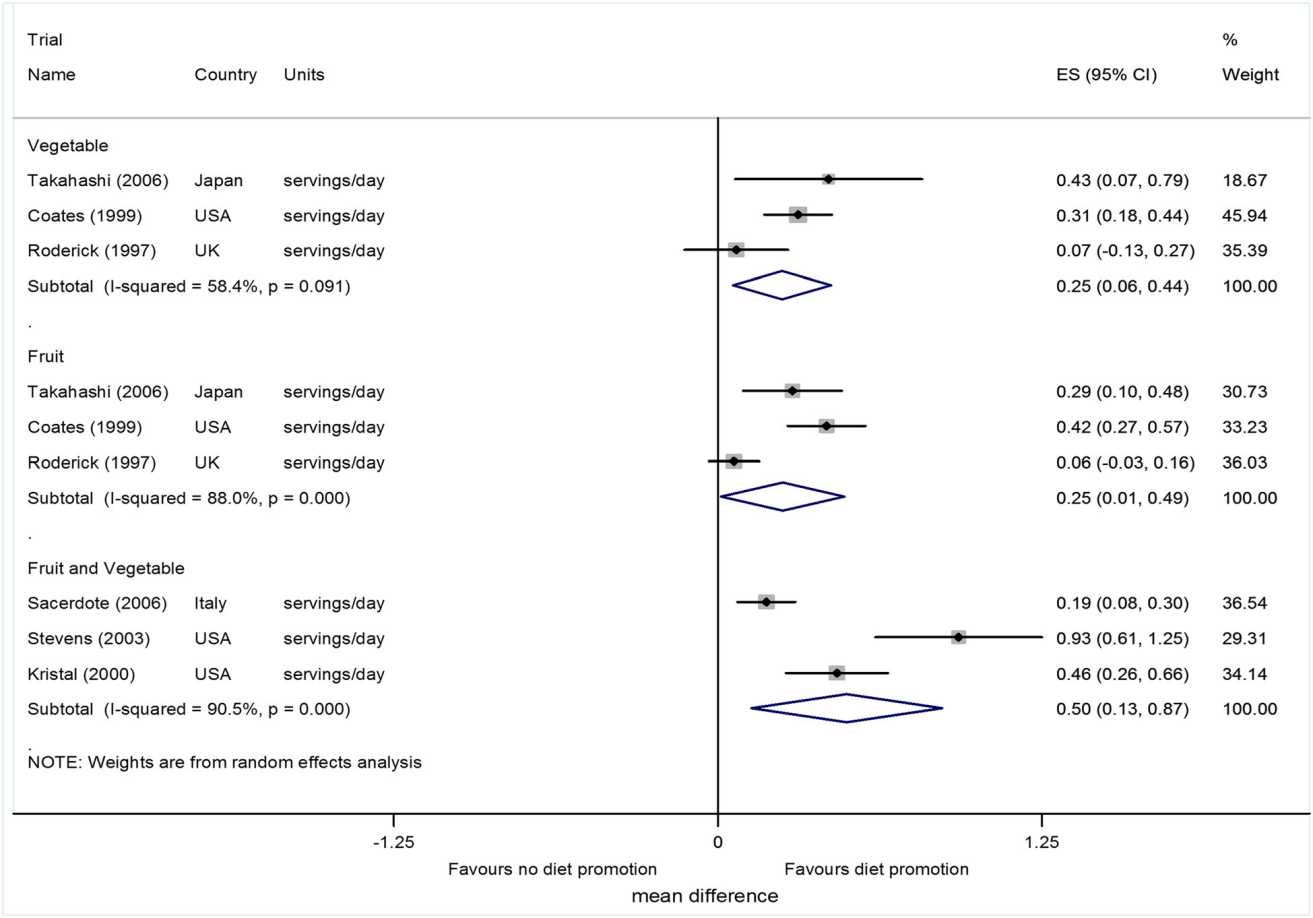
**Individual study and pooled effects of diet promotion on intakes of fruit, vegetables, and combined fruit and vegetable at 12 months.** ES = effect size, 95% CI = 95% confidence intervals.

Pooled analysis of three studies [27-29], which reported intervention effects on consumption of fruits and vegetables separately, showed a mean difference of 0.25 (95% confidence interval (CI) 0.01 to 0.49, p = 0.04) serving per day for fruit consumption. There was evidence of appreciable heterogeneity (I^2^ = 88.0%, p < 0.001) (Figure 2). Vegetable consumption was increased by 0.25 (0.06 to 0.44, p = 0.01) servings per day, with less evidence of heterogeneity among studies was I^2^ = 58.4%, p = 0.091 (Figure 2).Three studies [30-32] reporting intervention effects on consumption of fruit and vegetable combined together showed a pooled effect of 0.50 (0.13 to 0.87, 0.008) servings per day. There was evidence of substantial heterogeneity (I^2^ = 90.5%; p < 0.001) (Figure 2). The summary measure had a conservatively wide 95% confidence interval (0.13 to 0.87) for the pooled effect by use of the random effects model.

#### Intervention effect on fibre intake

**Figure 3:**
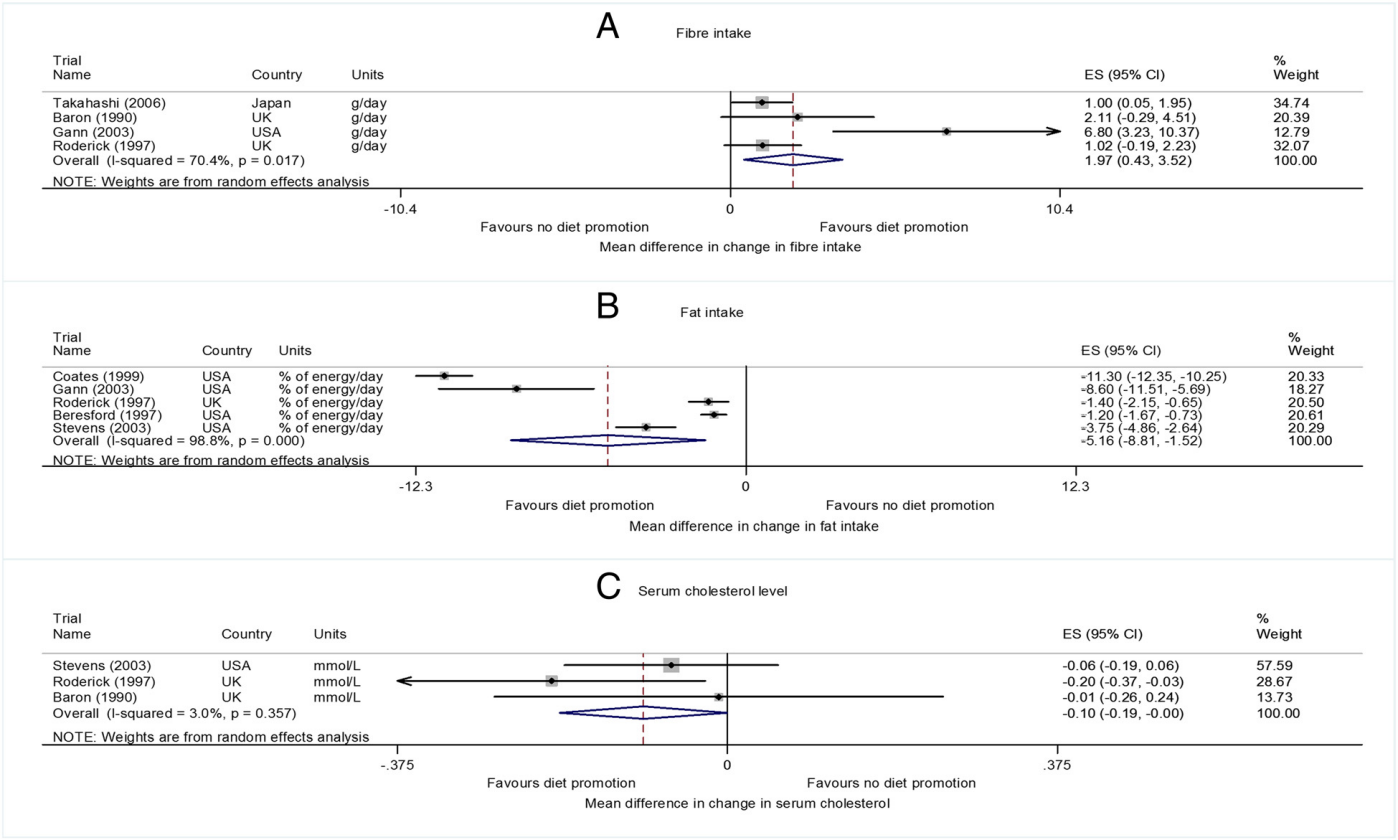
**Individual study and pooled effects of diet promotion on fiber and fat intake and serum cholesterol level at 12 months.** ES = effect size, 95%CI = 95% confidence intervals.

Six studies [27,29,33-36] reported the intervention effect on fibre intake, but two [34,35] of these studies were not included in the meta-analysis. The estimated intervention effects in the study [35] reporting the fibre intake in grams per 1000 kcal was 0.32 (SE 0.19) grams per 1000 kcal, whereas in the study [34] reporting the fibre intake score, the estimated intervention effect was -0.04 (SE 0.04), with a negative score indicating increased fibre intake. Pooled analysis of the remaining four studies showed evidence of increase of 1.97 (0.43 to 3.52, p = 0.012) grams of fibre consumption per day. There was some evidence of heterogeneity (I^2^ = 70.4%, p = 0.017) (Figure 3A).

#### Intervention effects on fat intake

Seven studies reported the effects of intervention on fat intake, but two [32,34] of these studies were not included in the meta-analysis. The estimated intervention effect in one [32] of these studies was -0.1(SE0.02) and in the other [34] study was -0.06 (SE 0.041), both expressed in scores scales with negative score indicating decreased fat intake. Pooled analysis from five studies [28,29,31,35,36] showed a mean decrease of 5.16% (95% CI -8.81 to -1.52, p = 0.005) in fat intake, expressed as percentage of total energy intake per day. There was heterogeneity between studies (I^2^ = 98.8%, p < 0.001) with one study [28] showing a very large change (Figure 3B).

#### Intervention effects on cholesterol

Dietary cholesterol intake was evaluated in one study [28] with a 79.2 mg (95% CI 61.9 to 96.5) more decrease in dietary cholesterol per day when compared to those not receiving the diet promotion intervention. Three studies [29,31,33] analysed the intervention effects in the serum cholesterol level. There was a 0.10 mmol/L (0 to 0.19 mmol/L, p = 0.049) more mean decrease in serum cholesterol level in people receiving the diet promotion intervention compared to the comparators (heterogeneity among studies I^2^ = 3%, p = 0.36) (Figure 3C).

#### The impact of intervention characteristics on observed effects

A descriptive analysis was implemented because the small number of included studies meant that it was not possible to conduct meta-regression analyses to formally test the impact of intervention characteristics on observed effect sizes. For fruit and vegetable intake, assessed either singly or in combination, and for serum cholesterol, effect sizes did not clearly increase with increasing participant contact. In contrast, for fibre and fat intake, the interventions with the most contacts clearly had the largest effects. None of the interventions that contributed data to pooled effect size estimates for fruit, vegetable or fibre intake were based on theory. However, for fruit and vegetable consumption considered together, interventions based on theory appeared to have larger effects than those not based on theory. In contrast, the most effective interventions for reducing fat intake were not based on theory, nor was there any clear benefit of using theory for reducing serum cholesterol. There was no clear relationship between the total number of intervention techniques used in an intervention and the effect sizes observed.

## Discussion

The results of this systematic review of randomised controlled trials suggest that moderately sustained but small effects on diet can be achieved through diet promotion interventions in primary care. The heterogeneity in observed effect sizes suggests that these interventions, despite all being delivered or deliverable in primary care, varied a great deal in their impact on behaviour. The studies employed a range of behaviour change techniques including one-to-one counselling along with variety of media with variable theoretical under-pinning. Incomplete reporting makes it difficult to establish how interventions were intended to achieve their effects. No study reported monitoring treatment fidelity, so it is unclear whether interventions were delivered as their designers planned. If interventions failed to change behaviour, was this because the components of the intervention were not effective? or because these were not successfully delivered?

### Strengths and limitations

Our review has several strengths compared with earlier reviews [14-17]. We estimated the long-term effectiveness of dietary interventions in primary care using only randomised controlled trials or cluster randomised trials with at least 12 months follow-up, where interventions were delivered mostly by health care professionals. In order to focus on a population approach to primary prevention, we did not include trials which included participants with existing chronic conditions or at high risk of diseases such as colorectal or breast cancer and participants with relatives or family members with chronic health problems linked to diet. We did not include trials implemented in the workplace or faith settings where characteristics of participants may be different from those attending the primary health care. We did not restrict the inclusion of trials on the basis of percentage loss to follow up which limited the evidence to trials with more adherent participants and we included two trials [29,34] in our review which were not included in the Cochrane review. The restriction of studies with more than 20% loss to follow up may introduce bias in the findings limited to trials with more adherent participants. We also explored the existing interventions in terms of behaviour change techniques used.

We acknowledge several limitations. Most of the included trials used self-reported measures of dietary change, and there are possibilities that intervention effects may have suffered responder bias. Furthermore, we cannot rule out the possibility of contamination in open trials which could have resulted in the exchange of information on diet modifications and may have modified outcomes in controls. Dietary interventions cannot be completely blinded; however, outcome assessors can be blinded to avoid the chance of selection bias in trials. Not blinding outcome assessors and trial personnel may have introduced bias in trials thereby overestimating the effectiveness of intervention. We restricted the inclusion of trials investigating the effects of multi-factorial interventions to avoid the potential confounding effect of other health promoting interventions. However, assessing effectiveness of only diet promotion interventions may have overestimated the effects in practice where diet promotion may run simultaneously with other health promotion activities. Where reported, most of the trial participants were white and had some college education, while few reported socio-economic status. We cannot confirm whether our results can be applied to populations with different ethnic, educational and socio-economic characteristics. We do not know whether it is cost-effective to implement diet promotion interventions with similar intensity as reported in the trials in the primary care general population. Participants with unknown previous exposures to health campaigns and media may increase the selection bias. We may have missed some unpublished or published trials.

### Comparison with other studies

A Cochrane systematic review [17] reported that diet promotion intervention in health care setting increased fruit and vegetable intake by 1.88 (95% CI1.07 to 2.70) servings per day; and irrespective of setting fruit intake alone increased by 0.67 (95% CI 0.007 to 1.28) servings per day and vegetable intake alone by 0.92 (95% CI0.34 to 1.49) servings per day, fibre intake increased by 6.51 (95% CI2.20 to 10.82) grams per day, in health care setting total dietary fat intake expressed as percentage of total calories fell by 5.38% (95% CI -7.84 to -2.92) and total blood cholesterol level reduced by 0.11 mmol/l (95% CI – 0.19 to -0.03). Effect sizes were generally considerably smaller in our study compared to the Cochrane review. The Cochrane review included studies with participants with chronic conditions, as well as participants at high risk of colorectal cancer and breast cancer. Studies were carried out in faith and work settings as well as in primary care. These differences in participant characteristics, resulting from differences in inclusion/exclusion criteria between our study and Cochrane review may explain these differences in results. Another review [16] published in 1998, reported 5.5% reduction in total blood cholesterol level; expressed in a different measurement unit than our study and we could not compare the changes in blood cholesterol with our results.

## Conclusions

Our review suggests that diet promotion interventions in the primary care population yield modest positive effects on diet intake over one year. Opportunities to promote dietary change may be taken by general practice based health care staff during patient general practice visits. Our findings should be interpreted with caution because of some of the limitations of the review and the included studies. The present studies may offer limited potential to inform the science of dietary behaviour change in primary care. For example, it is unclear whether interventions are ineffective or whether these are difficult to deliver in routine practice, possibly requiring greater training to deliver consistently. Our findings raise questions concerning whether a brief single diet counselling can be effective as more intensive diet promotion interventions; whether diet promotion interventions will have the same effect in minority ethnic groups and in a deprived general population; and whether the limited effects sizes are of long-term benefit in relation to resources used.

## Competing interests

The authors declare that they have no competing interests.

## Authors’ contributions

MCG conceived the study. All authors contributed to the design of the study. NB carried out the search, extracted the data, performed the analysis and prepared the first draft. AJW and NB extracted the data in terms of the behavioural theories and coded the interventions. MCG and NB cross checked the extracted data and analysis. All authors contributed to the revisions, read and approved the final manuscript.

## Pre-publication history

The pre-publication history for this paper can be accessed here:

http://www.biomedcentral.com/1471-2458/13/1203/prepub
